# Therapeutic efficacy of catalpol against apoptosis in cardiomyocytes derived from human induced pluripotent stem cells

**DOI:** 10.1186/s13568-020-00986-9

**Published:** 2020-03-20

**Authors:** Lingchao Yang, Xiangfei Feng, Yigang Li, Song Zhang, Yu Ying

**Affiliations:** Department of Cardiology, Xinhua Hospital, School of Medicine, Shanghai Jiao Tong University, 1665 Kongjiang Road, Shanghai, 200092 China

**Keywords:** Arrhythmia, Catalpol, Stem cells, Iridoid glycoside, Heart rhythm

## Abstract

Cardiac arrhythmia is an irregular heart rhythm that can lead to serious heart conditions and various organ disorders, and may cause sudden cardiac death. Catalpol belongs to the iridoid glycoside family and is highly abundant in *Rehmannia glutinosa* Libosch. The study included five groups such as group I (normal control), group II (treatment control), group III (low-dose treatment), group IV (medium-dose treatment) and group V (high-dose treatment). We investigated the therapeutic effects of catalpol on cardiac arrhythmia in human-induced pluripotent stem cells (iPSCs). Cell viability, lactate dehydrogenase (LDH) levels, lipid peroxidation, antioxidant activity, and caspase-3 and caspase-9 activities and protein levels were measured in normal control, treatment control, and treated (1, 10, and 100 µM) iPSC groups. Compared with the treatment control group, catalpol supplementation (1, 10, and 100 µM) increased iPSC cell viability by 7.5, 27.3, and 65.8%, respectively; reduced the LDH levels by 10.4, 31.3, and 75.2%, respectively; and reduced the lipid peroxidation levels by 7.7, 33.0, and 62.6%, respectively. The antioxidant levels were significantly higher in the treatment control group than in the normal control group. Catalpol (100 µM) reduced the caspase-3 and caspase-9 activities by more than 30% and increased expression of the corresponding proteins by more than 50%. These findings suggest that the naturally occurring iridoid glycoside catalpol is effective against aconitine-induced cardiac arrhythmia in iPSCs.

## Introduction

Cardiac arrhythmia an abnormal heart rhythm caused by irregular electrical activity (Benoist et al. 2012) that may lead to serious heart conditions and various organ disorders, and cause sudden cardiac death (Nattel and Harada 2014). Antiarrhythmic agents target the ion channels in the cardiomyocyte membrane, alter conduction velocity, and repress trigger activity to stabilize irregular electrical activity (Karagueuzian and Klein 2018). Although conventional therapeutic agents are effective against acute cardiac arrhythmia, they produce proarrhythmia and unwanted side effects (Asadi et al. 2014). Previous studies have shown that several natural drugs suppress acute arrhythmia (Brenyo and Aktas 2014) and acting through multiple pathways and target points (Li et al. 2017). Chuang et al. (2016) found that several natural drugs had strong antiarrhythmic effect with few side effects.

Catalpol is an iridoid glycoside belonging the iridoid glycoside family and is highly abundant in *Rehmannia glutinosa* Libosch. Catalpol has been demonstrated to have neuroprotective, anti-apoptotic, anti-inflammatory, and anti-oxidative properties in animal and cell culture studies (Jiang et al. 2015; Zheng et al. 2017). Hu et al. (2016) reported that catalpol inhibited apoptosis in cardiac myocytes via the mitochondrial-dependent caspase pathway. Pennacchio et al. (1997) showed that iridoid glycosides were cardioactive in rats, and Bi et al. (2018) found that catalpol protected against cardiac dysfunction in rats with induced myocardial infarction. Induced pluripotent stem cell (iPSC) lines derived from the somatic cells of patients and healthy individuals are crucial for biological studies involving drug discovery, mechanisms of heart disease, and heart development (Jeon et al. 2018). Thus, we investigated the therapeutic effects of catalpol on cardiac arrhythmia in human iPSCs.

## Materials and methods

Catalpol (molecular weight 362.33) and aconitine (molecular weight 645.74) were purchased from Sigma-Aldrich (Shanghai, China). 3-(4,5-dimethylthiazol-2-yl)-2,5-diphenyl tetrazolium bromide (MTT) assay kit (cell proliferation) (ab211091), lactate dehydrogenase (LDH) assay kit (colorimetric) (ab102526), anti-Caspase-3 antibody (ab4051) and anti-caspase-9 antibody (ab25758) were purchased from Abcam (UK).

### Cell culture

The human iPSC line was purchased from ATCC (ACS-1011, Manassas, VA, USA). Human iPSCs were cultured in suspension as described by Larijani et al. (2011). The cells were maintained in a CO_2_ incubator under standard culture conditions, and the medium was refreshed daily. Cardiomyocyte differentiation of the human iPSCs was induced using a protocol described by Pahlavan et al. (2018).

### Experimental groups

The study included five groups: Group I, normal control, human iPSCs incubated with dimethyl sulfoxide (DMSO) for 24 h; Group II, treatment control, human iPSCs incubated with 8 µM aconitine for 24 h; Group III, low-dose treatment, human iPSCs incubated with 8 µM aconitine and 1 µM catalpol for 24 h; Group IV, medium-dose treatment, human iPSCs incubated with 8 µM aconitine and 10 µM catalpol for 24 h; and Group V, high-dose treatment, human iPSCs incubated with 8 µM aconitine and 100 µM catalpol for 24 h.

### MTT assay

Human iPSCs were cultured in 96-well plates with 60,000 cells/well, and then divided into groups and treated with aconitine or DMSO and catalpol as indicated. At the end of the 24-h treatment period, the cells were incubated with MTT (0.5 mg/mL) for 4 h, after which the medium was removed carefully and the formazan crystals were dissolved in DMSO. The absorbance was measured at 570 nm. Cell viability is expressed as percentage of that in the control group (Debnath et al. 2015).

### LDH assay

Human iPSCs were cultured in 96-well plates with 60,000 cells/well, and then divided into groups and treated with aconitine or DMSO and catalpol as indicated. At the end of the 24-h treatment period, the medium was carefully removed and the LDH activity was measured, with the final absorbance measured at 450 nm (Soyingbe et al. 2018).

### Lipid peroxidation and antioxidant marker measurements

Lipid peroxidation in the human iPSC homogenate was determined as previously described (Lee et al. 2018). The concentrations of catalase, glutathione peroxidase (Gpx), superoxide dismutase (SOD), and reduced glutathione (GSH) in the human iPSC homogenate were determined according to previously described methods (Shaban et al. 2017).

### Caspase-3 and caspase-9 activities

Caspase-3 and caspase-9 activities were determined using a previously described method (Zhang et al. 2018). Briefly, the human iPSCs were cultured, divided into groups, and treated as described. At the end of the treatment, cells were harvested and lysed in caspase lysis buffer containing 5 mM DTT, 5 mM CHAPS, 50 mM HEPES and incubated with substrate in a solution containing 20 mM HEPES, 0.1% CHAPS, 5 mM DTT and 2 mM EDTA for 2 h at room temperature. The release of final product was analyzed using UV-1900i: UV–Vis Spectrophotometer (Shimadzu, Shanghai, China).

### Immunofluorescence

Immunofluorescent staining of caspase-3 and caspase-9 was performed according to Li et al. (2010). The cells were incubated with bovine serum albumin for 60 min, treated with anti-caspase-3 and anti-caspase-9 antibodies for 12 h, treated with horseradish peroxidase-conjugated secondary antibody for 60 min, and then signals were detected using fluorescence microscope (fluorescence microscope bx51, Olympus, Japan).

### Statistical analysis

Data are expressed as mean ± standard error of the mean (SEM). The groups were compared using Student’s *t*-tests and an analysis of variance (ANOVA). *P* values < 0.05 were considered to indicate statistical significance.

## Results

**Fig. 1:**
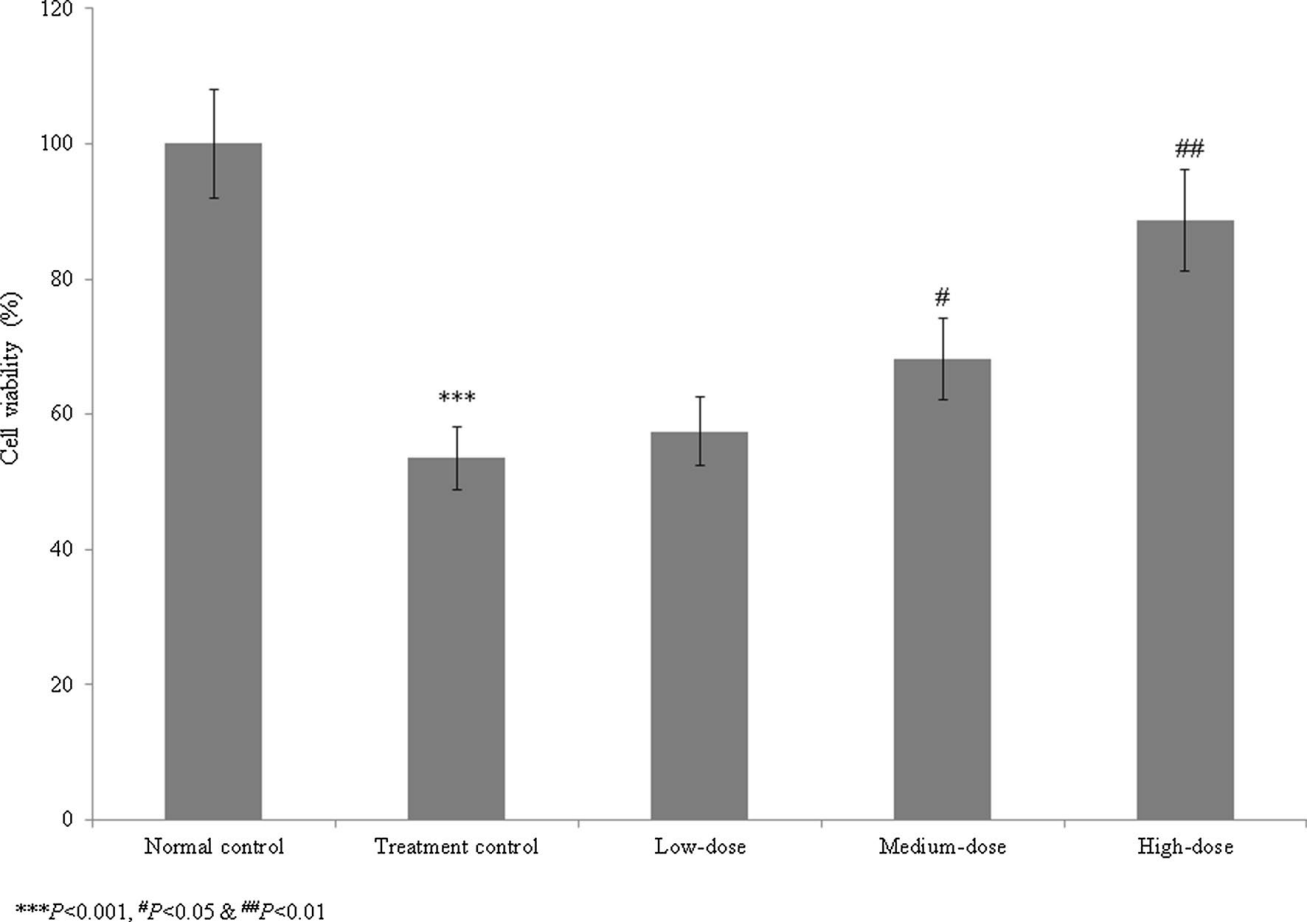
Effect of catalpol on the viability of human induced pluripotent stem cells with aconitine-induced cardiac arrhythmia. Catalpol supplementation increased cell viability by 7.5, 27.3, and 65.8% at 1, 10, and 100 µM concentrations, respectively. ***P < 0.001 vs. Group I; ^#^*P* < 0.05 and ^##^*P* < 0.01 vs. Group II

**Fig. 2:**
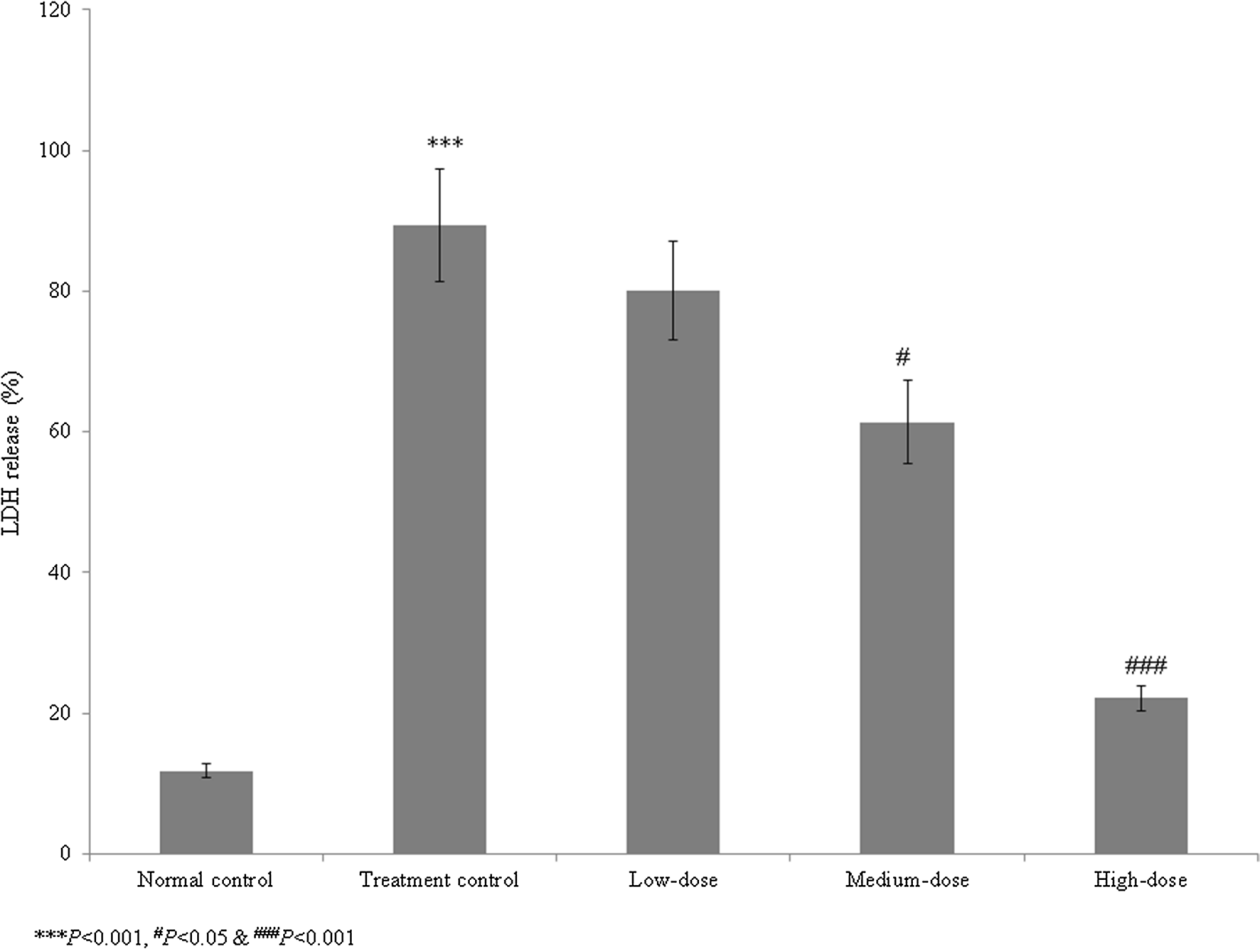
Effect of catalpol on the lactate dehydrogenase (LDH) levels in human induced pluripotent stem cells with aconitine-induced cardiac arrhythmia. Catalpol treatment reduced the LDH levels by 10.4, 31.3, and 75.2% at concentrations of 1, 10, and 100 µM, respectively. ***P < 0.001 vs. Group I; ^#^*P* < 0.05 and ^###^*P* < 0.001 vs. Group II

We assessed the therapeutic effects of catalpol on aconitine-induced cardiac arrhythmia in human iPSCs. The viability of the treatment control cells (Group II) was reduced to 46.5% that of the normal control cells (Group I). Catalpol treatment increased cell viability by 7.5, 27.3, and 65.8% at 1, 10, and 100 µM concentrations, respectively, compared to group II (*P* < 0.05, Fig. 1). The LDH level in the control group was 657.6% higher than that in the normal control group. Catalpol reduced the LDH levels by 10.4, 31.3, and 75.2% at 1, 10, and 100 µM, respectively, compared to the group II (*P* < 0.05, Fig. 2).

**Fig. 3:**
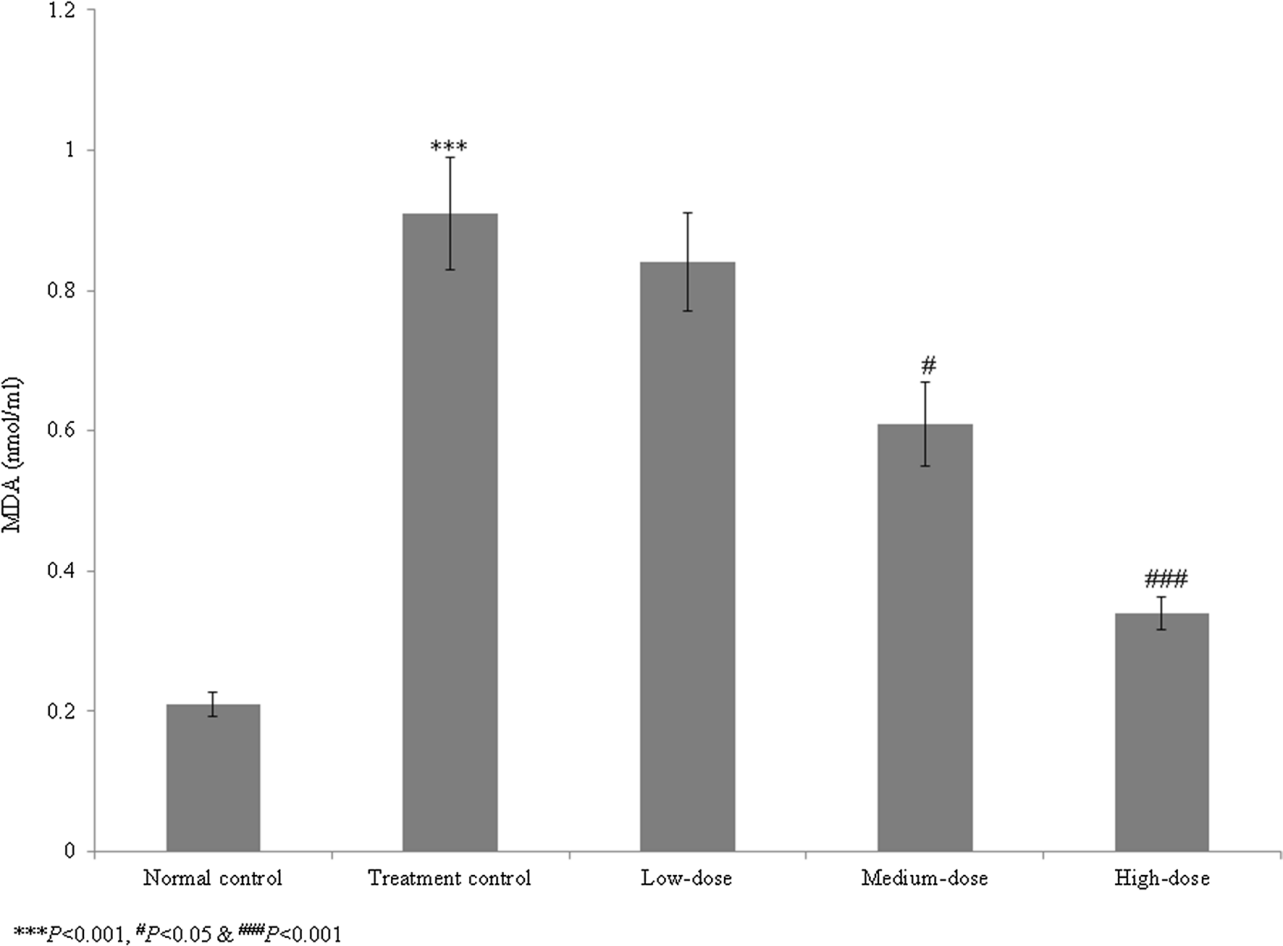
Effect of catalpol on lipid peroxidation in human induced pluripotent stem cells with aconitine-induced cardiac arrhythmia. Catalpol supplementation decreased lipid peroxidation by 7.7, 33, and 62.6% at 1, 10, and 100 µM respectively. ***P < 0.001 vs. Group I; ^#^*P* < 0.05 and ^###^*P* < 0.001 vs. Group II

In the control group, lipid peroxidation was significantly elevated, at 333.3% that in the normal control group. Catalpol treatment reduced lipid peroxidation by 7.7, 33.0, and 62.6% at 1, 10, and 100 µM, respectively, compared to group II (*P* < 0.05, Fig. 3). In the control, the levels of catalase, SOD, Gpx, and GSH were reduced significantly, by 69.2, 76.9, 67.6, and 71.4%, respectively (*P* < 0.05, Table 1). The high dose of catalpol (100 µM) increased the catalase, SOD, Gpx, and GSH levels more than 100% (*P* < 0.05, Table 1).

**Table 1:** Effect of catalpol supplementation on antioxidant markers in cardiac arrhythmia-induced pluripotent stem cells

Markers	Group I	Group II	Group III	Group IV	Group V
Catalase (U/mL)	11.7 ± 0.7	3.6 ± 0.2***	4.1 ± 0.3	6.8 ± 0.5^#^	10.4 ± 0.7^###^
SOD (U/mL)	317 ± 17	73 ± 5***	87.4 ± 6.2	167 ± 12^#^	293 ± 19^###^
Gpx (U/mL)	0.37 ± 0.02	0.12 ± 0.01***	0.16 ± 0.01	0.21 ± 0.01^##^	0.32 ± 0.01^###^
GSH (nmol/mL)	0.56 ± 0.04	0.16 ± 0.01***	0.21 ± 0.02	0.25 ± 0.02^##^	0.47 ± 0.03^###^

*** *P*<0.001, ^#^*P*<0.05, ^##^*P*<0.01 and ^###^*P*<0.001

**Fig. 4:**
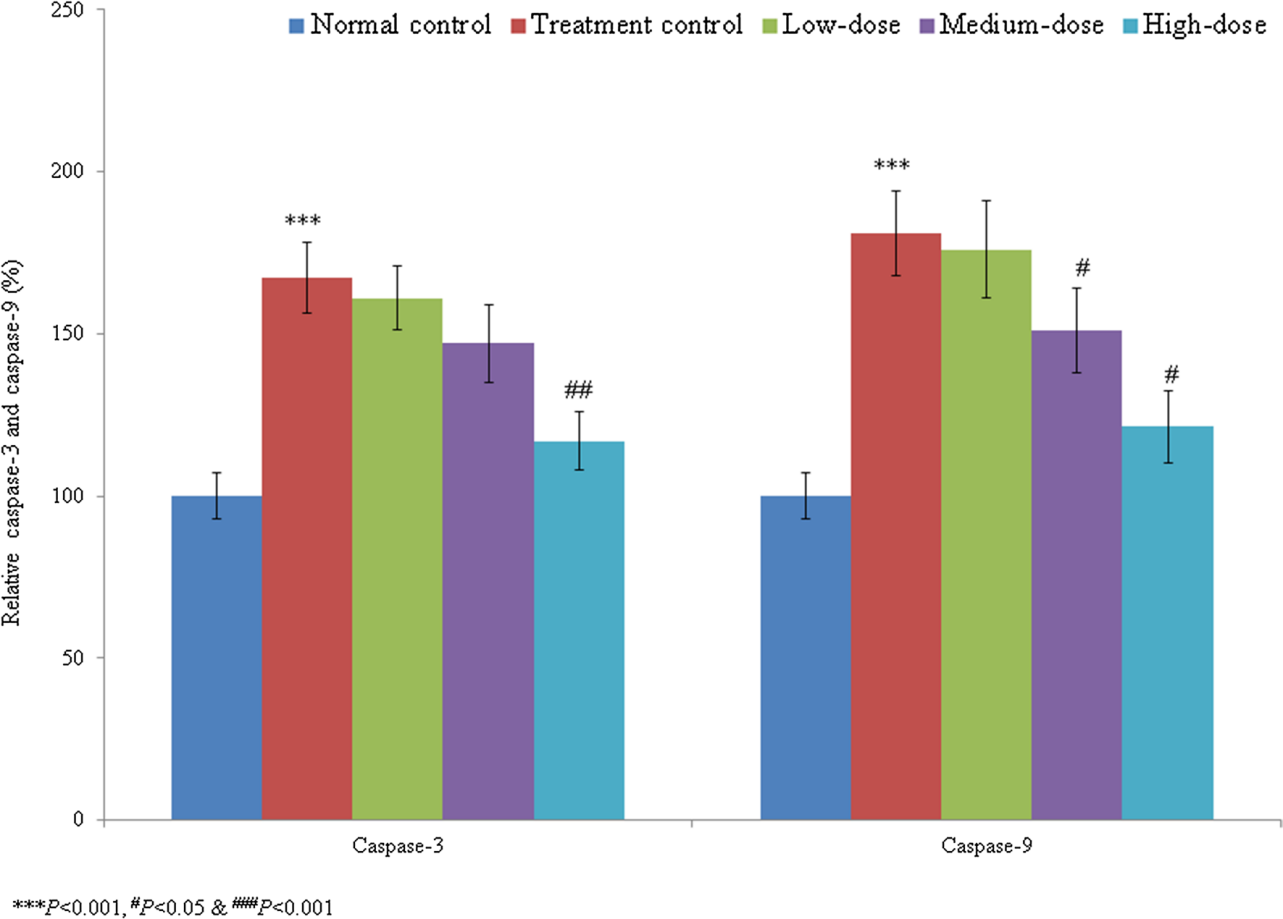
Effect of catalpol on caspase-3 and caspase-9 activities in human induced pluripotent stem cells with aconitine-induced cardiac arrhythmia. Catalpol treatment significantly reduced the caspase-3 and caspase-9 activities. ***P < 0.001 vs. Group I; ^#^*P* < 0.05 and ^###^*P* < 0.001 vs. Group II

**Fig. 5:**
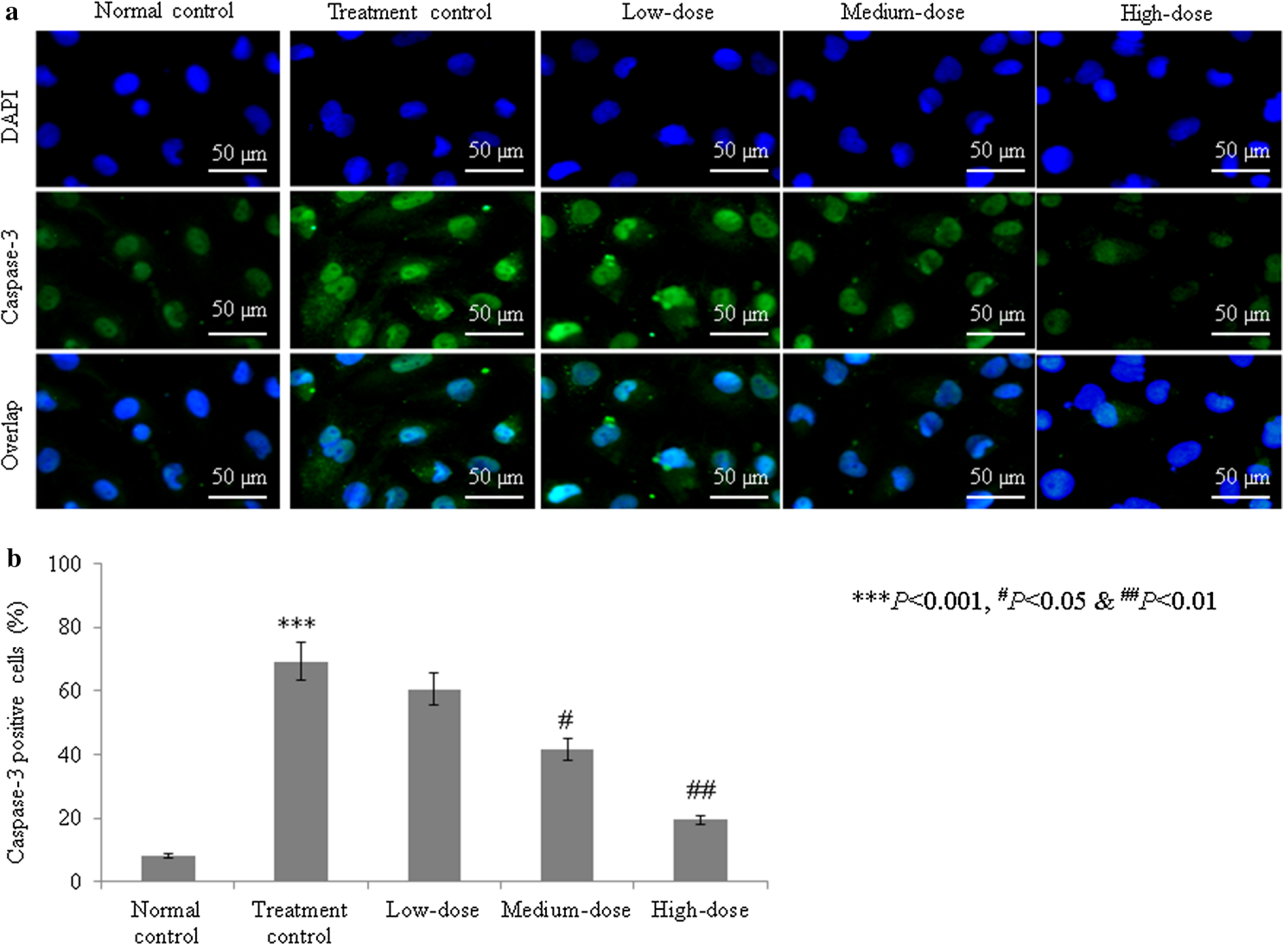
Effect of catalpol on caspase-3 expression in human induced pluripotent stem cells with aconitine-induced cardiac arrhythmia. Catalpol treatment reduced the percentage of caspase-3-positive cells by 12.7, 39.9, and 71.82% at 1, 10, and 100 µM concentrations, respectively. **a** Immunofluorescent images of caspase-3-positive cells. **b** Percentage analysis of caspase-3-positive cells. ***P < 0.001 vs. Group I; ^#^*P* < 0.05 and ^###^*P* < 0.001 vs. Group II. Scale bar is 50 µm

**Fig. 6:**
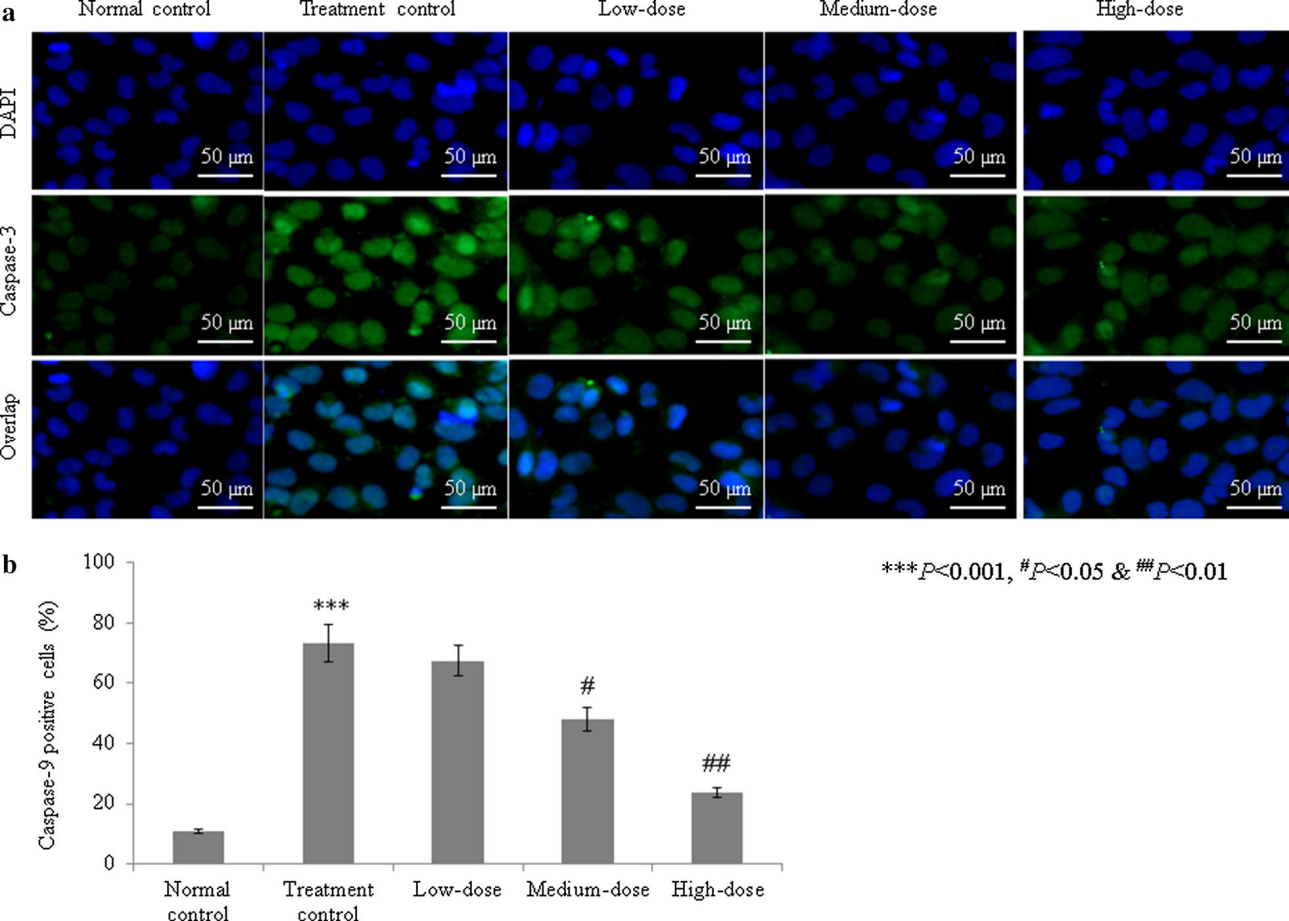
Effect of catalpol on caspase-9 expression in human induced pluripotent stem cells with aconitine-induced cardiac arrhythmia. Catalpol treatment decreased the percentage of caspase-9-positive cells by 7.9, 34.3, and 67.8% at concentrations of 1, 10, and 100 µM, respectively. **a** Immunofluorescent images of caspase-9-positive cells. **b** Percentage analysis of caspase-9-positive cells. ***P < 0.001 vs. Group I; ^#^*P* < 0.05 and ^###^*P* < 0.001 vs. Group II. Scale bar is 50 µm

The caspase-3 activity was significantly increased, by 67.4%, in the treatment control group compared with the normal control group (group I). Catalpol treatment reduced the caspase-3 activity by 3.8, 12.2, and 30.2% at 1, 10, and 100 µM concentrations, respectively (*P* < 0.05, Fig. 4). The caspase-9 activity was substantially increased, by 81%, in the treatment control group (group II) compared with the normal control group (group I). Catalpol treatment reduced the caspase-9 activity by 2.8, 16.6, and 33% at 1, 10, and 100 µM concentrations, respectively (*P* < 0.05, Fig. 4). The percentage of caspase-3-positive cells was markedly increased, by 755.6%, in the treatment control group compared with the normal control group. Catalpol treatment reduced the percentage of caspase-3-positive cells by 12.7, 39.9, and 71.82% at concentrations of 1, 10, and 100 µM, respectively (*P* < 0.05, Fig. 5). Similarly, the percentage of caspase-9-positive cells in the treatment control group markedly increased 584.1% compared with the normal control group. Catalpol supplementation reduced the percentage of caspase-9-positive cells 7.9, 34.3, and 67.8% at 1, 10, and 100 µM concentrations, respectively (*P* < 0.05, Fig. 6).

## Discussion

We assessed the therapeutic effects of catalpol on aconitine-induced cardiac arrhythmia in iPSCs. Cardiac arrhythmia is caused by irregular electrical activity in the heart rate or heart rhythm (Benoist et al. 2012). Antiarrhythmic agents target ion channels in the cardiomyocyte membrane, alter conduction velocity, and repress trigger activity to stabilize irregular electrical activity (Karagueuzian and Klein 2018). Although conventional therapeutic agents are effective against acute cardiac arrhythmia, they produce proarrhythmia and unwanted side effects (Asadi et al. 2014). Previous studies have shown that several natural drugs suppress acute arrhythmia (Brenyo and Aktas 2014), acting through multiple pathways and target points (Li et al. 2017).

Catalpol has been demonstrated to have neuroprotective, anti-apoptotic, anti-inflammatory, and anti-oxidative properties in animal and cell culture studies (Jiang et al. 2015). Catalpol reduces neointimal hyperplasia in diabetic rats (Lin et al. 2019). Liu et al. (2016) have reported that the catalpol treatment ameliorates diabetic atherosclerosis in diabetic rabbits. Researchers have reported that the catalpol reduced peroxynitrite formation and cardioprotective effects against ischemia/reperfusion insult (Huang et al. 2013). Hu et al. (2016) reported that catalpol inhibited apoptosis in cardiac myocytes via the mitochondrial-dependent caspase pathway. Pennacchio et al. (1997) showed that iridoid glycosides were cardioactive in rats, and Bi et al. (2018) found that catalpol protected against cardiac dysfunction in rats with induced myocardial infarction. We observed a marked reduction in the viability of human iPSCs in the treatment control group compared with the normal control group. However, catalpol treatment increased iPSC cell viability. The LDH levels were markedly increased in the treatment control group compared with the normal control group, whereas catalpol significantly reduced the LDH levels. These findings indicate that catalpol had a protective effect against aconitine-induced cardiac arrhythmia in human iPSCs.

Apoptosis plays a major role in the loss of cardiomyocytes (Kim and Kang 2010). To investigate the protective effect of catalpol on apoptosis, we measured the caspase-3 and caspase-9 activities in the groups after a 24-h incubation period. We found that catalpol supplementation decreased the activities of caspase-3 and caspase-9, confirming the therapeutic effect of catalpol against aconitine-induced apoptosis in iPSCs. Our findings suggest that catalpol, a naturally occurring iridoid glycoside, is effective against aconitine-induced cardiac arrhythmia in iPSCs.

## Data Availability

Corresponding author could provide the all experimental data on valid request

## References

[CR1] Asadi H, Yan B, Dowling R, Wong S, Mitchell P (2014). Advances in medical revascularisation treatments in acute ischemic stroke. Thrombosis.

[CR2] Benoist D, Stones R, Drinkhill MJ (2012). Cardiac arrhythmia mechanisms in rats with heart failure induced by pulmonary hypertension. Am J Physiol Heart Circ Physiol.

[CR3] Bi F, Xu Y, Sun Q (2018). Catalpol pretreatment attenuates cardiac dysfunction following myocardial infarction in rats. Anatol J Cardiol.

[CR4] Brenyo A, Aktas MK (2014). Review of complementary and alternative medical treatment of arrhythmias. Am J Cardiol.

[CR5] Chuang SF, Liao CC, Yeh CC (2016). Reduced risk of stroke in patients with cardiac arrhythmia receiving traditional Chinese medicine: a nationwide matched retrospective cohort study. Complement Ther Med.

[CR6] Debnath T, Ghosh S, Potlapuvu US (2015). Proliferation and differentiation potential of human adipose-derived stem cells grown on chitosan hydrogel. PLoS ONE.

[CR7] Hu LA, Sun YK, Zhang HS, Zhang JG, Hu J (2016). Catalpol inhibits apoptosis in hydrogen peroxide-induced cardiac myocytes through a mitochondrial-dependent caspase pathway. Biosci Rep.

[CR8] Huang C, Cui Y, Ji L, Zhang W, Li R, Ma L, Xing W, Zhou H, Chen B, Yu J, Zhang H (2013). Catalpol decreases peroxynitrite formation and consequently exerts cardioprotective effects against ischemia/reperfusion insult. Pharm Biol.

[CR9] Jeon H, Kim JY, Choi JK, Han E, Song CL, Lee J, Cho YS (2018). Effects of the extracts from fruit and stem of *Camellia japonica* on induced pluripotency and wound healing. J Clin Med.

[CR10] Jiang B, Shen RF, Bi J, Tian XS, Hinchliffe T, Xia Y (2015). Catalpol: a potential therapeutic for neurodegenerative diseases. Curr Med Chem.

[CR11] Karagueuzian HS, Klein U (2018). Wanted: class VI antiarrhythmic drug action; new start for a rational drug therapy. J Heart Health.

[CR12] Kim NH, Kang PM (2010). Apoptosis in cardiovascular diseases: mechanism and clinical implications. Korean Circ J.

[CR13] Larijani MR, Seifinejad A, Pournasr B, Hajihoseini V, Hassani SN, Totonchi M, Yousefi M, Shamsi F, Salekdeh GH, Baharvand H (2011). Long-term maintenance of undifferentiated human embryonic and induced pluripotent stemcells in suspension. Stem Cells Dev.

[CR14] Lee J, Cho YS, Jung H, Choi I (2018). Pharmacological regulation of oxidative stress in stem cells. Oxid Med Cell Longev.

[CR15] Li F, He Z, Shen J (2010). Apoptotic caspases regulate induction of iPSCs from human fibroblasts. Cell Stem Cell.

[CR16] Li J, Hu D, Song X, Han T, Gao Y, Xing Y (2017). The role of biologically active ingredients from natural drug treatments for arrhythmias in different mechanisms. Biomed Res Int.

[CR17] Lin CM, Wang BW, Fang WJ, Pan CM, Shyu KG, Hou SW (2019). Catalpol ameliorates neointimal hyperplasia in diabetic rats. Planta Med.

[CR18] Liu JY, Zheng CZ, Hao XP, Zhang DJ, Mao AW, Yuan P (2016). Catalpol ameliorates diabetic atherosclerosis in diabetic rabbits. Am J Transl Res.

[CR19] Nattel S, Harada M (2014). A trial remodelling and atrial fibrillation: recent advances and translational perspectives. J Am Coll Cardiol.

[CR20] Pahlavan S, Tousi MS, Ayyari M, Alirezalu A, Ansari H, Saric T, Baharvand H (2018). Effects of hawthorn (*Crataegus pentagyna*) leaf extract on electrophysiologic properties of cardiomyocytes derived from human cardiac arrhythmia-specific induced pluripotent stem cells. FASEB J..

[CR21] Pennacchio M, Syah YM, Ghisalberti EL, Alexander E (1997). Cardioactive iridoid glycosides from *Eremophila* species. Phytomedicine.

[CR22] Shaban S, El-Husseny MWA, Abushouk AI, Salem AMA, Mamdouh M, Abdel-Daim MM (2017). Effects of antioxidant supplements on the survival and differentiation of stem cells. Oxid Med Cell Longev.

[CR23] Soyingbe OS, Mongalo NI, Makhafola TJ (2018). In vitro antibacterial and cytotoxic activity of leaf extracts of *Centella asiatica* (L.) Urb, *Warburgia salutaris* (Bertol. F.) *Chiov* and *Curtisia dentata* (Burm. F.) C.A.Sm—medicinal plants used in South Africa. BMC Complement Altern Med.

[CR24] Zhang F, Cai L, Zhang J, Qi X, Lu C (2018). Aconitine-induced cardiac arrhythmia in human induced pluripotent stem cell-derived cardiomyocytes. Exp Ther Med.

[CR25] Zheng XW, Yang WT, Chen S, Xu QQ, Shan CS, Zheng GQ, Ruan JC (2017). Neuroprotection of catalpol for experimental acute focal ischemic stroke: preclinical evidence and possible mechanisms of antioxidation, anti-inflammation, and antiapoptosis. Oxid Med Cell Longev.

